# Correspondence

**Published:** 1890-01

**Authors:** 


NEW YORK, December 12, 1889.

Prof. T. Gaillard Thomas has just closed a series of six lectures on "Abortion" at the College of Physicians and Surgeons, and this is said to be his final appearance at the college with which he has been connected as professor of Gynæcology for a quarter of a century. The lecture-room was crowded with students and practitioners, who listened attentively to the eloquent remarks of the lecturer. Prof. Thomas was particularly forcible on the subject of "antisepsis," and related several stories from his private practice, and among others the following, which proves both interesting and instructive, since it illustrates a point which is very apt to be overlooked by the general practitioner: A few years ago he was summoned to the country to attend a lady who was suffering from puerperal septicæmia. There was no apparent cause for this condition as far as her attending physician could ascertain. Everything had been conducted with due regard to antisepsis, yet here was the fact of an infection staring him in the face. Prof. Thomas made a personal investigation, and noticed a closet adjoining the sick chamber. He examined it and found it to be one of those old-fashioned pan-closets which he jocosely designated as "whitened sepulchres," fair without and foul within. This was the solution of the mystery. The patient had been in the habit of going to this closet during her illness, and at each effort of defecation the genitals had soaked up, so to speak, the foul exhalatlons from this "chamber of horrors," as Prof. Thomas aptly described it. The moral to be learned from this is that during labor or abortion we should never permit the patient to sit on anything, when about to empty her bladder or bowels, except a commode or a vessel placed on the floor. The adoption of this simple rule will save the patient and physician a great deal of trouble.

At the last meeting of the Section of Laryngology of the Academy of Medicine, Dr. H. Holbrook Curtis read a paper on "The danger from the Indiscriminate Use of Cocaine."

Dr. Curtis thought that too little stress had been laid upon the question as to the responsibility involved in prescribing small doses of cocaine in acute coryzas and for the relief of conditions of nose and throat in which the discomfort is slight and other remedies are nearly as efficacious. Many persons are advised by their physicians to employ a 2 per cent. solution in the nose for the relief of an acute coryza or to overcome the feeling of oppression caused by congested turbinate bodies. At first the relief experienced is immediate, but the continuance of the drug gives rise to nasal stenosis, the overstimulation and contraction of the erectile tissues, giving way to permanent dilation due to vaso-motor paresis. Aside from this, the atomization of even a weak solution of cocaine in the nose is often attended by pleasurable constitutional effects, increased mental activity and general exhilaration, which induce the patient to resort to its use again, and lay the foundation for the cocaine habit. The author also stated that the indiscriminate use of cocaine among the laity has already introduced a condition which may be designated as the cocaine heart. The symptoms of this are a feeling of fullness and pain in the precordial region, later followed by enfeeblement of the heart action, palpitation, anæmia, and mental and bodily prostration, to relieve which the dose of the drug is increased and alcohol and tobacco in excess are usually also indulged in. This condition, together with the resulting nasal stenosis, usually brings the patient to the office of the specialist.

A lively discussion ensued after the reading of the paper, in which Dr. Koller, the discoverer of cocaine anæsthesia, who has recently been appointed a lecturer at the New York Polyclinic, participated. Dr. Beverly Robinson said he had not found cocaine to be a heart-depressant, but considered it a cardiac stimulant. Dr. Koller agreed in the main with the views expressed by Dr. Curtis, and stated that cocaine was certainly not to be trusted in the hands of the patient. He made it a rule never to allow the patient to have a prescription for cocaine. He believed that a weak solution applied to a mucous surface and allowed to remain for fifteen minutes, accomplished more than a stronger solution applied far a shorter time. Dr. Morris Asch concurred in the remarks as to the poisonous qualities of the drug in larger quantities, but had observed no deleterious effects from small doses. He noticed dizziness on only a few occasions. Dr. Agramonte reported two cases of convulsions in children from injection of small amounts of a 2 per cent. solution into the gums.

At a recent clinic Prof. R. F. Weir presented a woman suffering from ventral hernia. These hernias, the speaker remarked were a frequent cause of constipation in women, although often overlooked as a factor in this condition. This form of constipation is quite intractable to ordinary methods of treatment, but is relieved by the cure of the hernia. The diagnosis is readily made, the protruding mass conveying to the touch the sensation of a bag of beans rolling under the fingers. Dr. Weir advised a supporting bandage in this case, and deprecated operative interference in a woman past forty-five, since an operation was not entirely free from danger and was not curative in every case.

A very interesting discussion on the subject of "Stricture of the Urethra" took place at the surgical section of the Academy of Medicine last week. The paper of the evening was read by Dr. George E. Brewer, in which he gave a resume of one hundred and twenty operations for stricture of the urethra performed by himself. His conclusions were that stricture of anterior portion of the urethra, not further than five inches back, is best treated by internal urethrotomy performed always on the roof. Among the one hundred and twenty cases operated upon there were only three or four instances in which severe hemorrhage resulted, and this was readily controlled by the perineal crutch. Dr. Sturgis advocated divulsion as superior to urethrotomy in many cases, but this view found few adherents among the members present. He explained his preference on the ground that lacerated tissue was less apt to absorb septic material from the bladder than a clear cut wound. It was generally agreed among the members that in many cases of stricture the difficulty could be overcome by gradual dilation. Prof. Otis, however, stated that he defied any one to show in any surgical work published a single cure of stricture recorded from gradual dilation.

Under the able management of Dr. Wyeth, the Polyclinic is having a most successful winter session. The wards in both hospital buildings are crowded with patients, and the clinics are attended by large numbers of doctors from various sections of the country, with a large ropresentation from the South. Drs. T. D. McKown, of Columbus; C. D. Roy, of Atlanta; C. C. L. Rudicil, of Summerville, and L. M. Crichton, Atlanta, are taking courses at this institution. P. J. R.

